# IgG and IgA Antibodies Post SARS-CoV-2 Vaccine in the Breast Milk and Sera of Breastfeeding Women

**DOI:** 10.3390/vaccines10010125

**Published:** 2022-01-16

**Authors:** Federica Scrimin, Giuseppina Campisciano, Manola Comar, Chiara Ragazzon, Riccardo Davanzo, Mariachiara Quadrifoglio, Manuela Giangreco, Guglielmo Stabile, Giuseppe Ricci

**Affiliations:** 1 Department of Obstetrics and Gynaecology, Institute for Maternal and Child Health-IRCCS “Burlo Garofolo”, 34137 Trieste, Italy; federica.scrimin@burlo.trieste.it (F.S.); mariachiara.quadrifoglio@burlo.trieste.it (M.Q.); giuseppe.ricci@burlo.trieste.it (G.R.); 2 Department of Advanced Microbiology Diagnosis and Translational Research, Institute for Maternal and Child Health-IRCCS “Burlo Garofolo”, 34137 Trieste, Italy; giusi.campisciano@burlo.trieste.it (G.C.); manola.comar@burlo.trieste.it (M.C.); 3 Department of Medicine, Surgery and Health Sciences, University of Trieste, 34100 Trieste, Italy; ragazzon.chiara@gmail.com; 4 Neonatal Intensive Care Unit, Institute for Maternal and Child Health-IRCCS “Burlo Garofolo”, 34137 Trieste, Italy; riccardo.davanzo@gmail.com; 5 Epidemiology and Biostatistics Unit, Institute for Maternal and Child Health-IRCCS “Burlo Garofolo”, 34137 Trieste, Italy; manuela.giangreco@burlo.trieste.it

**Keywords:** COVID-19, vaccine, breastfeeding, antibodies, breast milk

## Abstract

The COVID-19 pandemic has carried massive global health and economic burden that is currently counteracted by a challenging anti-COVID-19 vaccination campaign. Indeed, mass vaccination against COVID-19 is expected to be the most efficacious intervention to mitigate the pandemic successfully. The primary objective of the present study is to test the presence of neutralizing anti-SARS-CoV-2 antibodies (IgA and IgG) in the breast milk and sera samples from vaccinated women at least 20 days after the complete vaccine cycle. A secondary aim is to compare the IgG antibodies level in maternal serum and breast milk. The third target is to evaluate the presence of the IgG antibodies in breast milk after several weeks from the vaccination. Finally, we collected information on the health status of infants in the days following maternal vaccination. Forty-two mothers were enrolled in the study. Thirty-six received the Pfizer/BioNTech vaccine, four the Astra Zeneca vaccine, one the Moderna vaccine and another woman Astra Zeneca in the first dose and Pfizer/BioNTech in the second dose. All 42 milk samples confirmed the presence of anti-SARS-CoV-2 IgG, and none showed IgA presence. Regarding the matched 42 sera samples, 41 samples detected IgG presence, with one sample testing negative and only one positive for seric IgA. None of the 42 infants had fever or changes in sleep or appetite in the seven days following the maternal vaccination. The level of IgG antibodies in milk was, on average, lower than that in maternal serum. According to our analysis, the absence of IgA could suggest a rapid decrease after vaccination even if frequent breastfeeding could favour its persistence. IgG were present in breast milk even 4 months after the second vaccine dose. Information on the immunological characteristics of breast milk could change mothers’ choices regarding breastfeeding.

## 1. Introduction

The COVID-19 pandemic has carried a massive global health and economic burden that is currently counteracted by a challenging anti-COVID-19 vaccination campaign [1]. Indeed, mass vaccination against COVID-19 is expected to be the most efficacious intervention to mitigate the pandemic successfully. Notably, reproductive-aged women account for a large proportion of the population, and their vaccination is considered essential to reach herd immunity.

Unfortunately, pregnant and breastfeeding subjects were not included in any of the trials for COVID-19 vaccines, causing persisting uncertainties and unsolved questions when vaccinating eligible adults [2].

This knowledge gap explains the lack of studies on the presence of anti-SARS-CoV-2 antibodies in the milk of vaccinated women and the adequacy of their human milk levels to provide passive immunity to newborns and infants.

The primary objective of the present study is to test the presence of neutralizing anti-SARS-CoV-2 antibodies (IgA and IgG) in the breast milk and sera samples from vaccinated women at least 15 days after the complete vaccine cycle. A secondary purpose is to compare the IgG antibodies level in maternal serum and breast milk.

The third aim is to evaluate the presence of the IgG antibodies in breast milk after several weeks from the vaccination.

Finally, we collected information on the health status of infants in the days following maternal vaccination.

## 2. Materials and Methods

The study sample includes lactating health workers and school workers who underwent COVID-19 vaccination. Women living in the Trieste area with physiological pregnancy and normal early postpartum were recruited from 1 February to 30 July 2021, by a Perinatal Study Group at the Institute for Maternal and Child Health—IRCCS Burlo Garofolo, Trieste.

The study was approved by the Internal Review Board of the Institute for Women and Child Health Burlo Garofolo in Trieste (IRB 19/2021). All participants were over 18 years of age and signed specific, informed consent.

The patients were all asymptomatic at the time of collection. Patients with chronic infections or who were or are currently suffering from cancer were excluded from the study. All the enrolled mothers and children underwent nasopharyngeal swabs for SARS-CoV-2 testing at the time of enrolment, one week before and one week after enrolment, constantly testing negative.

All patients underwent a single sample of serum and milk. After nearly complete breast expression, milk samples were obtained, including the foremilk and the hindmilk. Serum and breast samples were immediately sent to the laboratory for storage and analysis.

Breast milk samples were centrifuged at 800× *g* for 10 min at 4 °C in order to separate fat content from the cells. Detection of SARS-CoV-2 IgG neutralizing antibodies that recognized the receptor-binding domain (RBD) portion of the SARS-CoV-2 Spike protein was performed using an automatized semiquantitative enzyme-linked immunosorbent assay with 99% specificity and 96% sensitivity (HBelisa SARS-CoV-2, Eurospital Diagnostic, Trieste) [3]. The results of the HBelisa SARS-CoV-2 were displayed as OD (optical density), which indicated the levels of antibodies in the sample after comparison with positive and negative controls. The cut-off was determined by an internal reaction control. OD of the cut-off was used to calculate the OD index of the sample/OD of the cut-off.

When the OD of the sample/OD of the cut-off index was <0.8, it indicated that the sample had neutralizing antibodies against SARS-CoV-2. An index of ≥0.8 demonstrated that no neutralizing antibodies against SARS-CoV-2 were detected in the sample. The lower the index value (i.e., the closer to 0), the higher the concentration of antibodies distinguished in the sample [3].

Detection of SARS-CoV-2 IgA antibodies was performed employing an automatized qualitative enzyme-linked immunosorbent assay with 98.26% specificity and 100% sensitivity) (SARS-CoV-2 IgA, Eurospital Diagnostic, Trieste, Italy). After comparison with positive and negative controls, the test results were displayed as OD (optical density), symbolizing the presence/absence of antibodies in the samples. The cut-off was determined by an internal reaction control. OD of the cut-off was used to calculate the index (OD of the sample/OD of the cut-off).

An OD of the sample/OD of the cut-off index >1.4 showed that the sample presented neutralizing antibodies against SARS-CoV-2. An index between 1.2–1.4 indicated an uncertain result. An index <1.2 proved that no IgA antibodies against SARS-CoV-2 were observed in the sample. The test has been experimented and approved by the company for serum [3]. The test has been internally validated for milk. Preliminary testing was performed using milk from lactating women with a past infection of COVID-19 and milk from lactating healthy women (with no documented history of SARS-CoV-2 infection and negative swab prior to collection). All the samples were defatted by centrifugation and skim milk was harvested between the fat layer and the pellet. Different dilutions of the samples were tested. Results confirmed the presence of neutralizing antibodies in the milk of lactating COVID-19 women.

The samples were tested twice, presenting the mean of the two measurements.

### 2.1. Data Collection Method

An anonymous questionnaire investigated educational qualification, occupation, date of birth, history of prior SARS-CoV-2 infection, the timing of COVID-19 vaccine doses, Type of COVID-19 vaccine received, breastfeeding methods and the number of feedings per day. Moreover, medical history information was examined concerning any immune system diseases, immunoglobulin deficiencies, and the use of anti-inflammatory or immunosuppressive drugs.

Some questions in the questionnaire regarded the infant’s health in the days following vaccination: quality of sleep, meals, fever or other disturbances noted by the mothers.

### 2.2. Statistical Analysis

No formal sample size calculation was performed for this descriptive and exploratory study. All women who met the inclusion and exclusion criteria were recruited in the enrolment period.

Descriptive analysis of the sample was conducted using frequency with percentage for categorical variables and median with interquartile range (IQR) for continuous variables. The percentage of women with IgG antibodies in breast milk sample at least 15 days after the second dose of vaccine were calculated as the ratio between the number of women with a result <0.8 units/mL and the total number of enrolled women.

Wilcoxon signed ranks test was applied to evaluate the difference in the antibody response in the breast milk sample and the blood sample collected at least 15 days after the second vaccine dose. A *p*-value <0.05 was considered statistically significant.

## 3. Results

Forty-two subjects were enrolled in the study; their general characteristics have been reported in Table 1. They were all Caucasian. Thirty-six of them received Pfizer/BioNTech vaccine, four Astra Zeneca vaccine, one Moderna vaccine and another woman Astra Zeneca in the first dose + Pfizer/BioNTech in the second dose according to the Ministry of Health of Italy’s indications at the time of the study. Two individuals had developed COVID SARS infection in the 6 months before the collection with mild symptoms. Following the indications of the Italian Ministry of Health, these women received a single dose of the Pfizer/BioNTech vaccine. Twenty-four serum and milk samples were collected from our 42 patients between 20 days and 1 month after the second vaccine dose, 10 between 1 month and 2 months, and eight between three and four months to assess the presence of SARS-CoV-2 antibodies.

No patient showed severe adverse reactions to the vaccine, the most frequent side effects have been summarized in Table 1.

All the 42 milk samples attested the presence of anti-SARS-CoV-2 IgG, and no one showed IgA presence. Regarding the matched 42 sera samples, 41 samples revealed the presence of IgG with one sample testing negative (with an index value close to the positivity cut-off) and only one sample positive for seric IgA. The immunological situation of this patient was studied: total immunoglobulins were evaluated and found to be regular.

**Figure 1:**
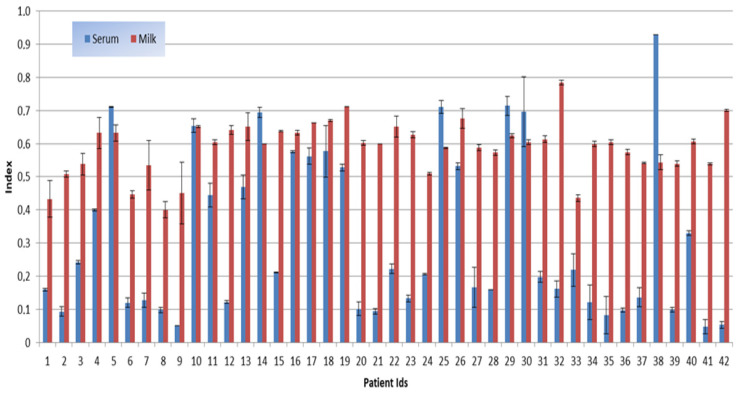
IgG antibodies in the observation period: 20 days to 4 months after the second dose of vaccine, in breast milk and serum. Data were obtained by the HBelisa SARS-CoV-2 neutralizing antibody detection kit (HB Healthcare corp.). If the **index is < 0.8**, the **sample is positive** for neutralizing antibodies to SARS-CoV-2. Each sample was tested twice. Data are presented as index value ± standard deviation.

In most cases (35/42, 84%), anti-SARS-CoV-2 IgG levels in serum were higher than in milk (Figure 1 and Table 2). All the sera and milk tested positive, regardless the time elapsed from the second dose of the vaccine, suggesting a lasting effect.

None of the 42 infants had fever or changes in sleep or appetite in the seven days following the maternal vaccination. One experienced a mild skin rash, which the mother interpreted as a reaction to sun exposure. Nobody went to the pediatrician. Eight mothers who otherwise would have stopped lactation (19.1%) after the vaccine, continued breastfeeding hoping to protect their babies from infection.

## 4. Discussion

One of the most important reasons for breastfeeding is breastmilk’s protection against infectious diseases [4]. It is plausible and expected [5] that human milk has a protective effect against COVID-19 due to its content of a-specific immuno-bioactive factors, such as lactoferrin, oligosaccharides, cytokines, and specific antibodies [6].

Generally, immunoglobulin A (IgA) is the most represented type in human milk, accounting for more than 90% of the total content of immunoglobulins. Only 5% of immunoglobulin A is absorbed, protecting infants’ guts until they produce their own IgA.

Following contact with SARS-CoV-2, antibodies are produced against different structural parts of the spike: receptor-binding domain (RBD), S1 and S2 subunits, and the nucleocapsid (N) protein. IgG, IgA, and IgM represent antibodies against RBD; they are neutralizing and induced by natural infection.

Our study showed the absence of IgA in serum and breast milk within 20 days in 41 women after the second vaccine dose, possibly dependent on a rapid decrease in the IgA antibody response as observed by other previous studies in COVID-19 convalescent individuals and vaccinated subjects [7,8].

Data from the COVID Milk—Power Milk study, including 2312 women, indicated that after SARS-CoV-2 natural infections, IgA antibodies in human milk remained detectable at least 10 months postinfection [9].

Pullen [10] observes antibody responses specific to SARS-CoV-2 in the serum and breast milk of infected women with a more dominant transfer of immunoglobulins A and M.

IgG would be functionally attenuated. The author observes the preferential transfer of antibodies capable of eliciting neutrophils phagocytosis and neutralization with respect to other functions. This would indicate a selective transfer of IgA and IgM accompanied by selected IgG subpopulations in the milk.

However, the presence and dynamics of antibodies after vaccination appeared different from those during infection.

In a prospective longitudinal study in 26 lactating women, Juncker [11] observed a biphasic response with SARS-CoV2 specific immunoglobulin A, starting to increase 5–7 days after the first vaccine dose. Mainly, IgA showed a biphasic response, the first 1–2 weeks after the first dose of the vaccine and the second accelerated response one week after the second inoculation, reaching 86% positivity. The population was studied up to 15 days after the second dose.

Perl [12] observed in 84 women that mean levels of anti-SARS-CoV-2-specific IgA antibodies in the breast milk were significantly elevated 2 weeks after the first vaccine (61.8% of samples tested positive), increasing to 86.1% 1 week after the second dose. Average levels remained elevated in 65.7% of samples tested two weeks after the vaccination cycle.

Unlike the studies just mentioned, which investigated the first 15 days after the second vaccine dose, we analyzed the presence of immunoglobulins in our sample from 20 days after the second dose of the vaccine to 4 months later. The different sampling times could justify the absence of Ig A in our patients’ milk and serum samples. Furthermore, the faster decline of IgA could be related to IgA’s natural kinetics. Indeed, as seen for influenza vaccination, intramuscular vaccination elicited a more substantial IgG presence in human milk than a mucosal exposure during natural infection where the IgA plays a pivotal role [13]. Our data found confirmation in other studies such as that of Gray et al. that presented an increase in immunoglobulin G, but not immunoglobulin A, in maternal blood and breastmilk after the second vaccine dose (boost dose) [14]. Another study recently published by Young et al. agreed with our data by showing that vaccination was associated with a uniform IgG-dominant response, while IgA increased in human milk only after the first dose reducing in a second time and this result was confirmed by other studies [15,16].

A previous study demonstrated a strong positive correlation between secretory SARS-CoV-2 IgA concentrations and lactation duration [17]. We found in our sample a single woman with IgA positive in serum but not in milk 3 months after the vaccine. She used to breastfeed more than eight times per day for a long time.

Focusing on IgG response, we evaluated the antibody trend in serum and breast milk in the months following vaccination.

Our data documented that IgG neutralizing antibodies were detectable regardless the time elapsed from the second dose of the vaccine both in serum and breast milk (10 women were tested between 1 and 2 months after the second dose of vaccine and eight patients were tested between 3 and 4 months). These results were consistent with Esteve Palau et al. [18], who tested the breast milk of 33 women vaccinated with the novel mRNA-based Pfizer-BioNTech vaccine two weeks after the first and second dose and four weeks after the second dose only. He found that breast milk IgG(S1) levels increased after the second dose and were positively associated with corresponding serum levels.

Our study also proved that in 84% of cases, the antibody level remained higher over time in maternal serum than in milk

Similarly, Perl et al. [11] demonstrated that anti-SARS-CoV-2-specific IgG antibodies raised in the fourth week, reaching 91.7% of positive samples and further increasing to 97% at weeks 5 and 6 from the first vaccine.

Jakuszko et al. [19] confirmed our results, showing that IgG levels grew in breast milk only after the second inoculation. The highest concentrations of breast milk antibodies were observed around day 29, with a decrease around day 43 from the first dose. Notably, the duration of IgG in milk was longer than that of IgA. Charepe analyzed the relationship between the duration of breastfeeding and IgG/IgA levels in milk finding a moderately positive correlation with IgG [16].

However, if confirmed, the predominance of IgG in breast milk is particularly relevant since its crucial role in neonatal immunity [20,21].

Our health data for the 42 infants documented that no mother detected adverse effects that warranted referral to the pediatrician. One mother had the impression that her son was more nervous than usual, a child had mild flu symptoms in the days following the maternal vaccination, and a baby had a mild trunk rash.

The administration of the mRNA vaccine to the lactating woman was recommended by scientific societies [22,23,24,25,26,27]. COVID vaccine was immunogenic [6,28] and lacked evidence of harm for the vaccinated nursing mother and her breastfed infant [29]. Our data confirmed these indications.

The safety of COVID vaccination during pregnancy (in the second and third trimester according to Italian ministerial guidelines) or breastfeeding it is currently a topic of great scientific and social interest, particularly for women employed in jobs at a higher risk of COVID transmissions, such as health care and school education. A complex set of reasons has often caused a delay in the acceptance or rejection of vaccines despite the availability of vaccination services (i.e., vaccine hesitancy).

Women needed clear, evidence-based information on the compatibility between the COVID vaccine and pregnancy/breastfeeding to decide how to protect themselves and their nursed infants best even after starting day-care, once the mothers were back to work. In our population, eight women (19.1%), who were considering stopping breastfeeding due to returning to work, continued breastfeeding after learning of the neutralizing antibodies present in milk.

The data relating to the health of infants, although limited in number and time, are the result of replies to a questionnaire and a form of active supervision, performed in the 38% of cases by medical doctor mothers and in the 19% of cases midwives mothers

Our study was one of the few in the literature on vaccinated women tested after several weeks from the second dose of the vaccine.

The limitation of our study was represented by a limited sample, with white, healthy and working patients.

## 5. Conclusions

The content of anti-SARS-CoV-2 antibodies in breast milk after infection and vaccination suggested a possible protective effect for the nursing infant. The level of IgG antibodies in serum was, on average, higher than that in maternal milk.

After vaccination, IgA decayed rapidly even if frequent breastfeeding could favor its persistence.

IgG were detectable regardless the time elapsed from the second dose of the vaccine bon in serum and in breast milk.

Information on the immunological characteristics of breast milk can change mothers’ choices regarding breastfeeding. In our series, seven women who would have stopped breastfeeding with the resumption of work, decided to extend breastfeeding once our data was known.

Our sample was too small to give us indications of possible adverse effects of the vaccine on infants; however, our little series was reassuring. Responses relating to the effects of vaccines of mothers on nursing infants would require active surveillance of this population through questionnaires or periodic interviews for a long time.

This should be the subject of other studies.

## Figures and Tables

**Table 1:** Characteristics of the populations.

Variable	*n* = 42
**Age, Median (IQR)**	34.5 (33.0–39.0)
**Education, *n* (%)**	
High school diploma	4 (9.5)
Degree	33 (78.6)
Postgraduate	1 (2.4)
PhD	4 (9.5)
**Occupation, *n* (%)**	
Medical doctor	16 (38.0)
Nurse-midwife	8 (19.1)
Teacher	8 (19.1)
Other	10 (23.8)
**Comorbidity, *n* (%)**	7 (16.7)
Basedow disease	1 (2.4)
Behcet disease	1 (2.4)
Hypothyroidism	2 (4.8)
Hashimoto’s thyroiditis	3 (7.2)
**Drugs in the last 10 days, *n* (%)**	6 (14.3)
Antihistamines	2 (4.8)
Anti-inflammatory	1 (2.4)
Other	3 (7.2)
**Previous COVID-19 infection, *n* (%)**	2 (4.8)
**Vaccine type**	
Pfizer/BioNtech	35 (83.3)
Astrazeneca	4 (9.5)
Moderna	2 (4.8)
Mixed	1 (2.4)
**Days between collection and the 2nd dose of vaccine, median (IQR)**	22 (16–46)
**At least one adverse event, *n* (%)**	20 (47.6)
Injection site pain	11 (26.2)
Headache	4 (9.5)
Pyrexia	9 (45.0)
Myalgia	9 (45.0)
Ostealgia	2 (4.8)
Arthralgia	2 (4.8)
Allergic reaction	0 (0.0)
Asthenia	12 (28.6)
Chills	3 (7.2)
**Vaccine influence on breastfeeding, *n* (%)**	8 (19.1)
**Number of milk feed, median (IQR)**	5 (4–7)
**OD milk, median (IQR)**	0.8 (0.7–1.2)
**OD serum, median (IQR)**	0.3 (0.2–0.8)
**Index milk, median (IQR)**	0.6 (0.5–0.6)
**Index serum, median (IQR)**	0.2 (0.1–0.5)
**New-born age in months, median (IQR)**	11.5 (8–17)
**Days between delivery and vaccine, median (IQR)**	319.5 (111.0–486.0)
**Adverse events in the child within 7 days after the vaccine**	
Restlessness	1 (2.4)
Cold	1 (2.4)
Skin rash	1 (2.4)
**Infant’s attendance of kindergarten, *n* (%)**	7 (16.7)

**Table 2:** Comparison of antibody level in serum and milk (Wilcoxon test).

	N	Min	25%	Median	75%	Max	Wilcoxon Rank Sign Test *p*-Value
**OD milk**	42	0.37	0.67	0.81	1.20	1.60	
**OD serum**	42	0.05	0.15	0.27	0.78	1.68	
**Diff OD**	42	−1.26	−0.71	−0.46	−0.20	0.58	<0.0001
**Index milk**	42	0.40	0.54	0.60	0.64	0.78	
**Index serum**	42	0.05	0.12	0.20	0.53	0.93	
**Diff Index**	42	−0.65	−0.44	−0.32	−0.14	0.39	<0.0001

## Data Availability

The original contributions presented in the study are included in the article, further inquiries can be directed to the corresponding author.
